# Ratiometric fluorescent probe with AIE property for monitoring endogenous hydrogen peroxide in macrophages and cancer cells

**DOI:** 10.1038/s41598-017-07465-5

**Published:** 2017-08-04

**Authors:** Yong Liu, Jing Nie, Jie Niu, Fangfang Meng, Weiying Lin

**Affiliations:** 1 Institute of Fluorescent Probes for Biological Imaging, School of Chemistry and Chemical Engineering, School of Materials Science and Engineering, University of Jinan, Jinan, Shandong 250022 P.R. China; 2 School of Chemical Engineering & Technology, China University of Mining and Technology, Xuzhou, Jiangsu 221116 P.R. China

## Abstract

Hydrogen peroxide (H_2_O_2_) plays a key role in the progression of human illnesses, such as autoimmune and auto-inflammatory diseases, infectious diseases, diabetes, and cancer, etc. In this work, we have discribed a novel probe, **TPE-TLE**, which remarkably displayed AIE property and ratiometric fluorescence emission profiles in the presence of H_2_O_2._ This ratiometric fluorescent probe with AIE property exhibits outstanding features such as the well-resolved emission peaks, high sensitivity, high selectivity, low cytotoxicity, and good cell-membrane permeability. These excellent attributes enable us to demonstrate the ratiometric imaging of endogenously produced H_2_O_2_ in macrophages and cancer cells based on the novel ratiometric probe with AIE property for the first time. By comparing two kinds of cells, it is firstly found that cancer cells should contain much more endogenous H_2_O_2_ than macrophages. We expect that **TPE-TLE** will be useful fluorescent platform for the development of a variety of ratiometric fluorescent probes with AIE property to achieve unique biological applications.

## Introduction

In 2001, the novel phenomenon of aggregation-induced emission (AIE) was first found by Tang’s group^1^. AIE materials show very weakly fluorescence features in solution state and became intense in the aggregated state^2, 3^. The above unique finding has become a new method to tackle the aggregation-caused quenching (ACQ) of conventional chromophores and has shown significant academic value and promising applications in cell imaging^4^, fluorescent sensors and bio-probe materials^5, 6^. Herein, we describe the development of a novel ratiometric fluorescent probe with AIE property for ratiometric monitoring bioactive small molecule in the living system.

Reactive oxygen species (ROS) are formed as a natural by-product of the normal metabolism of oxygen and play a key role in cell signaling and homeostasis^7–9^. Generation of excessive ROS is involved in the pathogenesis of various diseases such as cardiovascular disease, cancer and neurological disorders^10–12^. Hydrogen peroxide (H_2_O_2_), a major ROS, exhibits relatively mild reactivity and has attracted intense interest^13–17^. Because it appears to be involved in signal transduction by reversible oxidation of proteins^18^. It has been known for many years that H_2_O_2_ plays a major role in the progression of human illnesses, such as autoimmune and auto-inflammatory diseases, infectious diseases, diabetes, mutagenesis and, perhaps most importantly, cancer^19–23^. Consequently, the search for a method that can be used for monitoring H_2_O_2_ has always been attractive and challenging.

Owing to the transient nature of H_2_O_2_, fluorescent probes, which generally display high sensitivity and can be used to determine spatial and temporal distributions in live specimens, are particularly appealing tools for the detection of ROS and related metabolites^24^. Most of these probes are intensity-based type, and tend to be interfered by the variations in excitation intensity, probe concentration, etc. To alleviate these problems, a number of ratiometric fluorescent H_2_O_2_ probes have been developed^25, 26^. However, up to present, there have been no reports on an AIE material for ratiometric sensing and imaging endogenous H_2_O_2_ in living cells (Table S1). Thus, developing ratiometric fluorescent probe with AIE property is very important due to significant academic value and biological applications of AIE materials. Thus, the goal of our work is to design a ratiometric fluorescent probe with AIE property for ratiometric detecting H_2_O_2_ in different cell lines.

In this work, we described a novel ratiometric H_2_O_2_ fluorescent probe with AIE property for the first time. This ratiometric fluorescent probe with AIE property exhibits outstanding features such as the well-resolved emission peaks, high sensitivity, high selectivity, low cytotoxicity, and good cell-membrane permeability. These features were promising this novel AIE material can be succesfully applied for ratiometric imaging endogenous H_2_O_2_ in living RAW 264.7 macrophages and cancer cells HepG2. Compared to RAW 264.7 macrophages, we firstly found that cancer cells HepG2 should contain much more endogenous H_2_O_2_.

## Results and Discussion

### Preparation of probe

**Figure 1:**
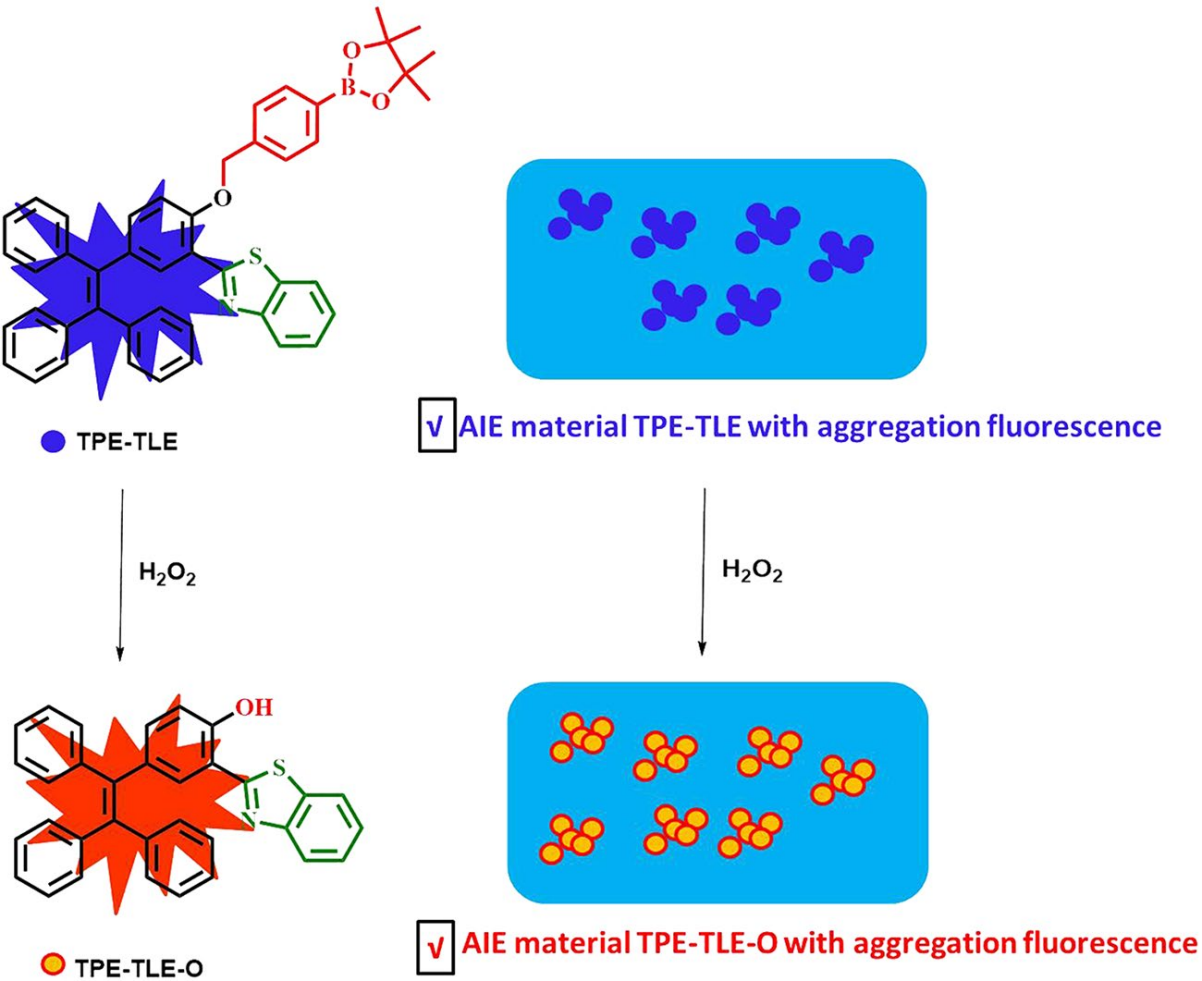
The sense mechanism of the probe **TPE-TLE** to H_2_O_2._

As we all know, tetraphenylethene (TPE) is an archetypal AIE luminogen^27^. On the basis of our interest on AIE material^28^, we further exploited the unique application of this class of dyes by rational sturctural modifications. As shown in Fig. 1, we introduced a thiazole group on the TPE-core to afford the novel AIE material TPE-TLE-O. On the other hand, modification of a H_2_O_2_ site such as a borate moiety on the TPE-TLE-O gave the new compound **TPE-TLE**, which exhibited AIE character distinguishing from TPE-TLE-O. We envision that both materials exhibit distinct aggregation fluorescence signals.

For the AIE material **TPE-TLE**, in the presence of H_2_O_2_, the oxidation reaction of H_2_O_2_ to the borate moiety will provide TPE-TLE-O, which will decrease aggregation fluorescence signal of **TPE-TLE** to induce aggregation fluorescence signal of TPE-TLE-O. Therefore, we envisioned that AIE material **TPE-TLE** may be suitable for ratiometric imaging endogenous H_2_O_2_ in living systems.

**Figure 2:**
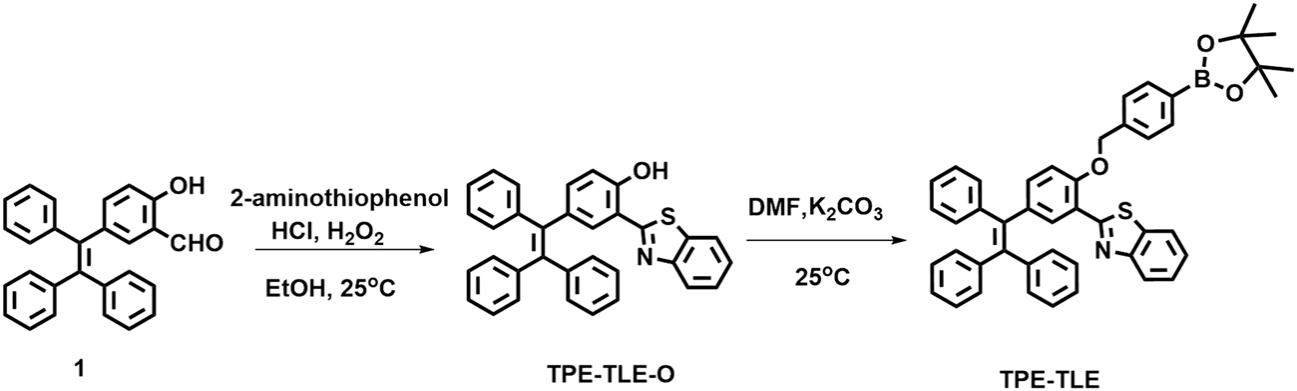
The synthetic routes of the compound **TPE-TLE**.

Chemical synthesis of the compound **TPE-TLE** was accomplished in a total of two steps (Fig. 2). The compound TPE-TLE-O was prepared by the ring-closing reaction between compound **1** and 2-aminothiphenol. **TPE-TLE** was synthesized by a substitution reaction. The ^1^H NMR, ^13^C NMR, and H RMS were given in the Supporting Information.

### Optical properties in various solvents

**Figure 3:**
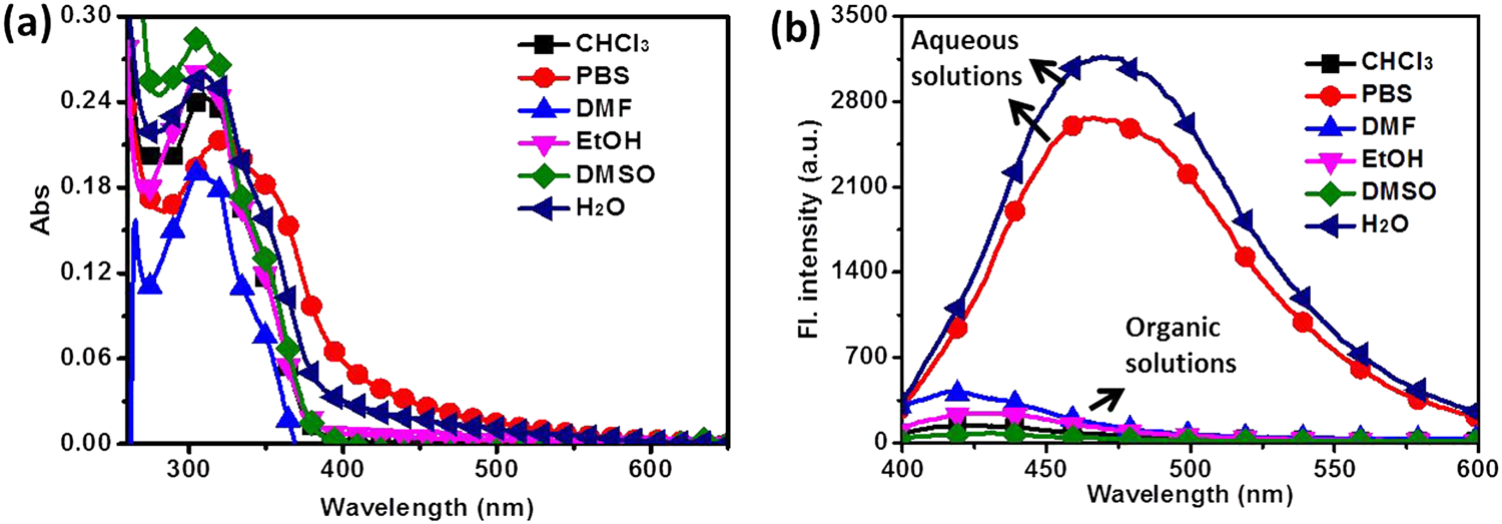
(**a**) The UV–vis absorption and (**b**) fluorescence spectra of **TPE-TLE** in aqueous solutions and organic solvents. [**TPE-TLE]** = 5.0 μM.

Optical properties of probes were basic properties of compounds. These properties provided clues for speculating its optical applications. Thus, with **TPE-TLE** in hand, we first evaluated its absorption and fluorescence profile in aqueous solutions and organic solutions. As shown in Fig. 3 and Table S2, our studies revealed that **TPE-TLE** had absorption and emission peak at 305 nm and 475 nm in aqueous solutions; In organic solutions, the absorption and emission peak of probe emerged at 305 nm and 420 nm. The results indicated that this probe had larger Stokes shift in aqueous solutions than organic solvents. Moreover, the probe exhibited higher fluorescence quantum yield (*Φ* = 0.61) in pure water system than organic solvents (*Φ* = 0.02 in EtOH). As anticipated, the aggregation of the compound **TPE-TLE** may affect optical physical properties of compound in pure water system. Introduction of a thiazole group on the TPE-core will obtain a novel AIE material **TPE-TLE**.

### AIE property

**Figure 4:**
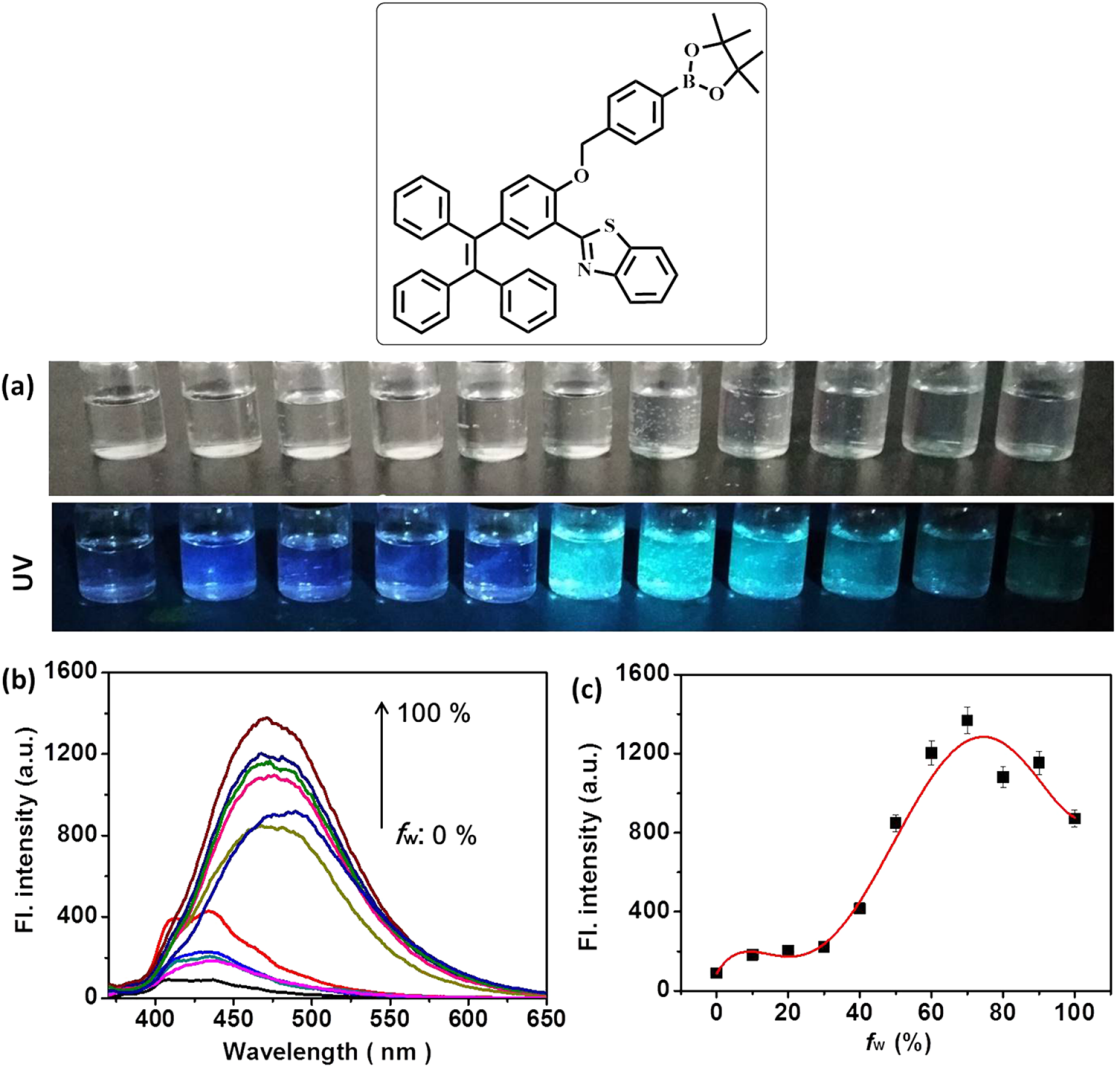
(**a**) Photographs of **TPE-TLE** in water/DMF mixtures taken under nature light and UV illumination; (**b**) Fluorescence spectra of **TPE-TLE** in water/DMF mixture; (**c**) Plots of maximum emission intensity of **TPE-TLE** in water/DMF mixtures with different *f*_w_ values. [**TPE-TLE**] = 5.0 μM. λ_ex_ = 365 nm. The error bars represent standard deviation ( ± S.D.).

To verify the compound **TPE-TLE** whether was a fluorescent material with AIE property, we investigated optical properties of **TPE-TLE** in the distinct polar environments. We set out to investigate the fluorescence behavior of **TPE-TLE** in water/DMF (*f*_w_) mixtures. Firstly, we prepared eleven organic solutions of **TPE-TLE** with different water fraction (*f*_w_); Photographs of **TPE-TLE** in water/DMF mixtures were taken under UV illumination (Fig. 4a). The above result demonstrated that the blue fluorescence signal of **TPE-TLE** gradually became strong when *f*_w_ increased from 0% (*Φ* = 0.11) to 100% (*Φ* = 0.61). Secondly, we systematically changed *f*_w_ value, and further measured the emission spectra of **TPE-TLE** in water/DMF mixtures (Fig. 4b). Fluorescence trend present gradually strong with increasing of *f*_w_ values (Fig. 4c). Fluorescence characteristics of **TPE-TLE** were consistent with photographs of **TPE-TLE** in the distinct polar environments. Thus, we predicted that the compound **TPE-TLE** should be a fluorescent material with AIE property. The optical studies of **TPE-TLE** revealed that rational structural modifications on the TPE-core could exhibit AIE character.

**Figure 5:**
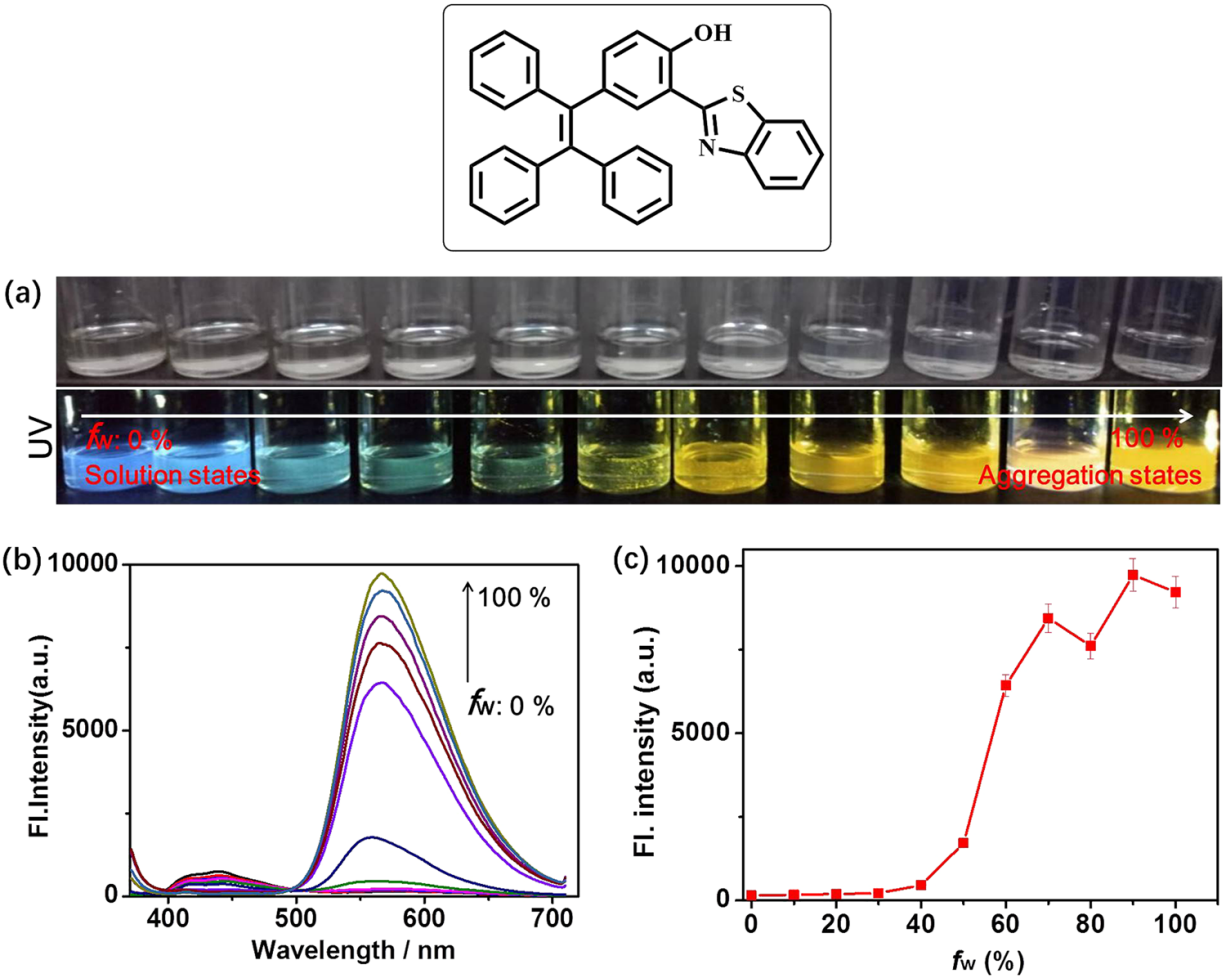
(**a**) Photographs of TPE-TLE-O in water/DMF mixtures taken under nature light and UV illumination; (**b**) Fluorescence spectra of TPE-TLE-O in water/DMF mixture; (**c**) Plots of maximum emission intensity of TPE-TLE-O in water/DMF mixtures with different *f*_w_ values. [**TPE-TLE**] = 5.0 μM. λ_ex_ = 365 nm. The error bars represent standard deviation ( ± S.D.).

In preparation of probe, we have envisioned that the oxidation reaction of H_2_O_2_ to the borate moiety will provide TPE-TLE-O in the presence of H_2_O_2_, which will decrease aggregation fluorescence signal of **TPE-TLE** to induce aggregation fluorescence signal of TPE-TLE-O. The TPE-TLE-O should be an AIE material distinguishing from **TPE-TLE**. To prove the above assumption, we investigated optical properties of TPE-TLE-O in the distinct polar environments. As shown in Fig. 5, photographs and fluorescence characteristics of TPE-TLE-O in water/DMF mixtures indicated that the compound TPE-TLE-O was an AIE material distinguishing from **TPE-TLE**.

The AIE material **TPE-TLE** should be suitable for ratiometric detecting H_2_O_2_ based on an aggregation emission method.

### Ratiometric responses of probe TPE-TLE to H_2_O_2_

**Figure 6:**
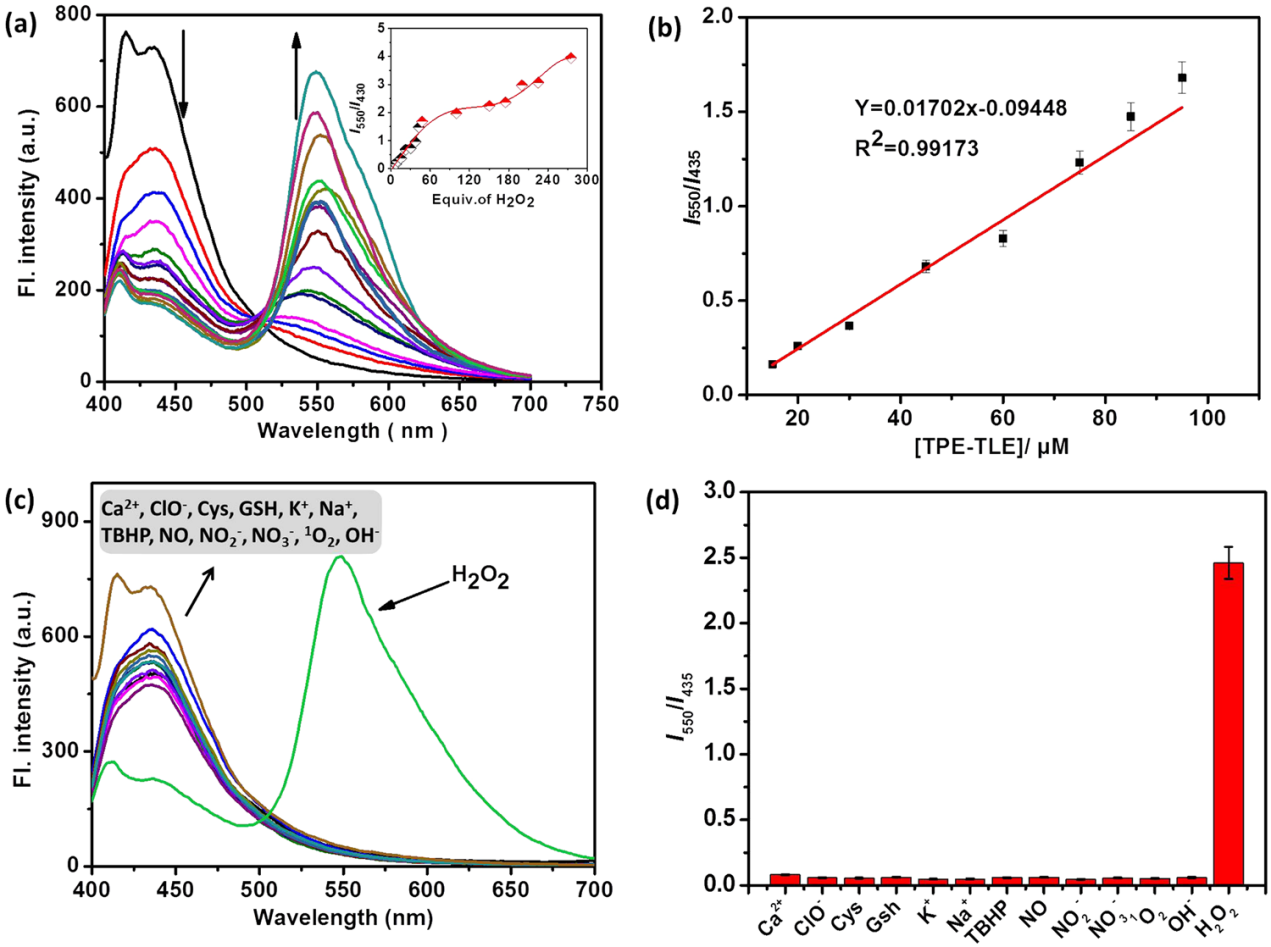
(**a)** Fluorescence spectra of **TPE-TLE** (5.0 μM) in pH 7.4 PBS buffer solution (containing 50% DMF) with the addition of H_2_O_2_ (0–200 equiv.) Inset: the fluorescence intensity changes at *I*_550_/*I*_435_ of **TPE-TLE** with the amount of H_2_O_2_. (**b**) Normalized response of the fluorescence signal by changing the concentration of H_2_O_2._ λ_ex_ = 380 nm. (**c**) Fluorescence spectra of **TPE-TLE** (5 μM) in the presence of various relevant analytes. (**d**) Fluorescence responses of **TPE-TLE** (5 μM) at 550 nm in the presence of various relevant analytes. The concentrations of the representative analytes are: amino acids, 1 mM; GSH, 2 mM; cations and anions, 3 mM; reactive oxygen and nitrogen species, 0.2 mM. The error bars represent standard deviation ( ± S.D.).

To examine whether probe **TPE-TLE** could ratiometric detect H_2_O_2_, **TPE-TLE** (5 μM) was treated with H_2_O_2_ in PBS buffer solution (containing 50% DMF), and further studying the progress of the reaction by fluorescence titration experiment. As shown in Fig. 6a, in the absence of H_2_O_2_, the probe **TPE-TLE** exhibited no visible variations in the ratios of emission intensities at 435 nm, suggesting that **TPE-TLE** was stable in the assay conditions. When increasing concentrations of H_2_O_2_ were introduced, the fluorescence spectra of probe **TPE-TLE** exhibited significant changes. The intensity of the emission maximum at 435 nm was gradually decreased with the simultaneous appearance of a new red-shifted emission band centered at 550 nm. The red-shift in the excitation spectra of the TPE with the increase H_2_O_2_ is due to intramolecular charge-transfer (ICT)^29, 30^. Moreover, the ratios of emission intensities at 550 and 435 nm (*I*_550_/*I*_435_) displayed a large increase from 0 to 4.25 (Fig. 6a, inset). Furthermore, a new fluorescence emission peak was observed at 550 nm. This result suggests that a new compound was formed during the titration process. The reaction product TPE-TLE-O was confirmed using ^1^H NMR (Fig. S1). The AIE material **TPE-TLE** was capable of detecting H_2_O_2_ by an aggregation emission method.

Sensitivity was an important criterion for developing highly sensitive fluorescence imaging agents^31^. Mainly reason was that the pathophysiologic ally relevant concentrations of H_2_O_2_ were in the low to medium micro molar concentrations^32^. We further investigate sensitive of probe in order to prove whether **TPE-TLE** can detect H_2_O_2_ in intercellular environment. As shown in Fig. 6b, the detection limit of the probe was 6.0 μM, indicating that the probe **TPE-TLE** exhibited highly sensitive to H_2_O_2_ by an aggregation emission method. We expect that the probe **TPE-TLE** was able to detect low micro molar concentrations of H_2_O_2_ and possessed potentially applicate in medical and biological.

To demonstrate the selectivity of **TPE-TLE** to H_2_O_2_, **TPE-TLE** was treated with a wide variety of cations, anions, and oxidants, and then fluorescence spectra were further measured. As shown in Fig. 6c and d, the probe **TPE-TLE** exhibited a significant red-shift of the fluorescence emission spectra in the presence of H_2_O_2_. Other representative species such as Ca^2+^, Na^+^, K^+^, ClO^−^, NO_2_^−^, NO_3_^−^, OH^−^, ^1^O_2_, H_2_O_2_, NO, TBHP, Cys, and GSH were added to **TPE-TLE** solutions. H_2_O_2_ elicited a large ratiometric signal with *I*_550_/*I*_435_. By contrast, other species induced a very low ratiometric response with *I*_550_/*I*_435_. This clearly indicated that the probe **TPE-TLE** had an excellent selectivity to H_2_O_2_ over the other analytes. The AIE material **TPE-TLE** was capable of highly selective detecting H_2_O_2_ by an aggregation emission method. In addition, the time-dependent fluorescence intensity changes from the probe **TPE-TLE** to H_2_O_2_ were further studied. As shown in Fig. S2, the probe **TPE-TLE** to H_2_O_2_ revealed that the reaction can be completed about 3 h.

We conducted another experiment condition to show that the probe can detect H_2_O_2_ in a ratiometric fashion *in vitro*. To examine the ratiometric fluorescence response of probe **TPE-TLE** to H_2_O_2_, the probe was titrated with different equiv. of H_2_O_2_ in 90% PBS buffer solution (containing 10% DMF). As shown in Fig. 4a and inset, the free probe displayed an emission peak with maximum at around 450 nm. By contrast, upon addition of H_2_O_2_, the intensity of the emission peak at 450 nm gradually decreased with the simultaneous appearance of a new blue-shifted emission band centered at 560 nm. This result was similar with response of probe **TPE-TLE** to H_2_O_2_ in 50% PBS buffer solution (containing 50% DMF). Moreover, as shown in Fig. S4b, the probe **TPE-TLE** exhibited higher sensitivity (Detection limit: 1 μM) in PBS buffer solution (containing 10% DMF) than 50% PBS buffer solution (containing 50% DMF). Furthermore, selectivity experiment indicated that the probe has high selectivity for H_2_O_2_ in 10% PBS buffer solution (containing 10% DMF) (Fig. S4c and d). The above results demonstrated that probe **TPE-TLE** could ratiometric detect H_2_O_2_ in 10% PBS buffer solution (containing 10% DMF).

We investigated influence of the probe **TPE-TLE** to H_2_O_2_ under different pH conditions. The results revealed that the pH value of solution had a notable influence on the probe response to H_2_O_2_. As shown in Fig. S4, in the presence of H_2_O_2_, the fluorescence intensity at 550 nm of probe response to H_2_O_2_ had no changes with the value increase of pH in the range of 5.0–8.0. In the absence of H_2_O_2_, the free probe **TPE-TLE** exhibited weak fluorescence intensity in the wide pH range. The above results demonstrated that the **TPE-TLE** can respond well to H_2_O_2_ without dramatic influenced by pH.

### Living cell imaging studies in RAW264.7 macrophages

In order to be useful as ratiometric imaging agents, ratiometric fluorescent probe should have low cytotoxicity. Thus, we investigated the potential toxicities of **TPE-TLE** against a representative cell: RAW264.7 macrophages. The living cells were incubated with various concentrations of the new ratiometric probe for 24 h, and then the cell viability was determined by the standard 3-(4,5-dimethylthiazol-2-yl)-2,5-diphenyltetrazolium bromide (MTT) assays; The results indicated that the ratiometric probe do not exhibit marked cytotoxicity (Fig. S5).

**Figure 7:**
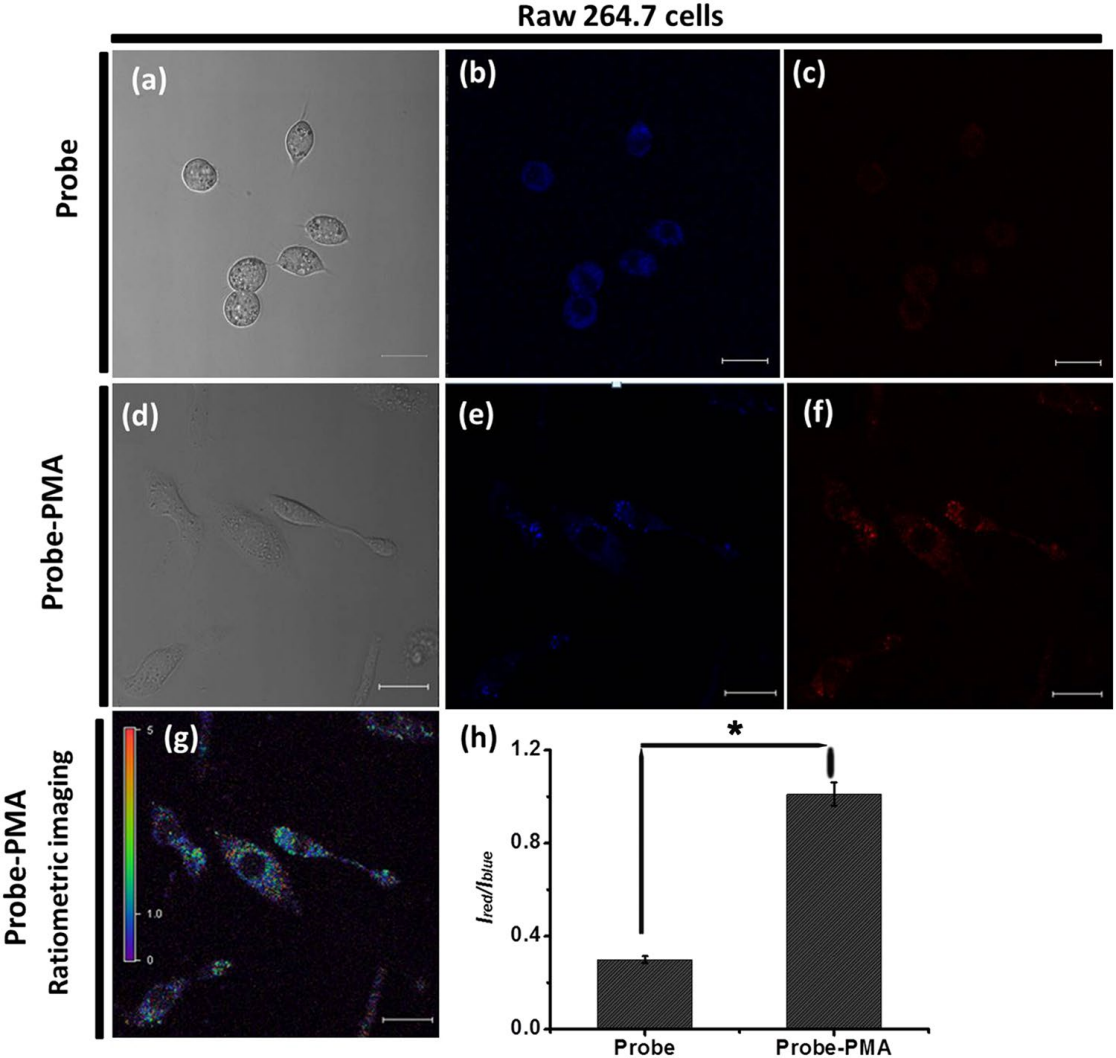
Image of endogenous H_2_O_2_ in RAW 264.7 cells untreated and treated with PMA. (**a**) Bright-field image of cells untreated with PMA, (**b**) Images of cells untreated with PMA in the blue emission channel (λ_ex_ = 405 nm, λ_em_ = 425–475 nm); (**c**) Images of cells untreated with PMA in the red emission channel (λ_ex_ = 405 nm, λ_em_ = 570–620 nm); (**d**) Bright-field images of cells treated with PMA; (**e**) Images of cells treated with PMA in the blue emission channel (λ_ex_ = 405 nm, λ_em_ = 425–475 nm); (**f**) Images of cells treated with PMA in the red emission channel (λ_ex_ = 405 nm, λ_em_ = 570–620 nm); (**g**) Ratiometric image of cells untreated with PMA; (**h**) The ratio of emission intensities profiles in RAW 264.7 cells untreated and treated with PMA. Scale bar = 20 μm. Statistical analyses were performed with a Student’s *t*-test (*n* = 4). *P < 0.001 and the error bars represent standard deviation ( ± S.D.).

As we know, H_2_O_2_ widely exists in RAW 264.7 macrophages and plays key role in apoptosis and oxidative stress^33^. Thus, to prove the capability of the probe **TPE-TLE** for H_2_O_2_ ratiometric fluorescence imaging in living cells, **TPE-TLE** (8 µM) was incubated with living RAW 264.7 macrophages for 0.5 h at 37 °C, and then confocal imaging was carried out. As shown in Fig. 7, cells emitted a strong blue fluorescence signal and weak red fluorescence in the blue (Fig. 7b) and red channels (Fig. 7c), respectively. Because endogenous H_2_O_2_ will be produced by stimulating RAW264.7 macrophage cells using 12-myristate-13-acetate (PMA). Then **TPE-TLE** (8 µM) was incubated with living RAW 264.7 macrophages treated with PMA for 0.5 h at 37 °C, and further achieved cells imaging. Imaging results demonstrated that cells displayed a slight blue emission (Fig. 7e) and a strong red emission (Fig. 7f), consistent with the H_2_O_2_-induced ratiometric fluorescent response *in vitro*. Moreover, we further obtained ratiometric fluorescence imaging picture of both channels in living RAW 264.7 macrophages (Fig. 7g). The above results indicated that the probe **TPE-TLE** was capable of ratiometric imaging endogenous H_2_O_2_ in living RAW 264.7 macrophages by an aggregation emission method. The ratios of emission intensities profiles further prove that the **TPE-TLE** emitted stronger fluorescent intensity in living RAW 264.7 macrophages treated with PMA than RAW 264.7 macrophages untreated with PMA (Fig. 7h). This result was agreement with H_2_O_2_-induced ratiometric fluorescent response *in vitro*.

### Living cell imaging studies in cancer cells HepG2

**Figure 8:**
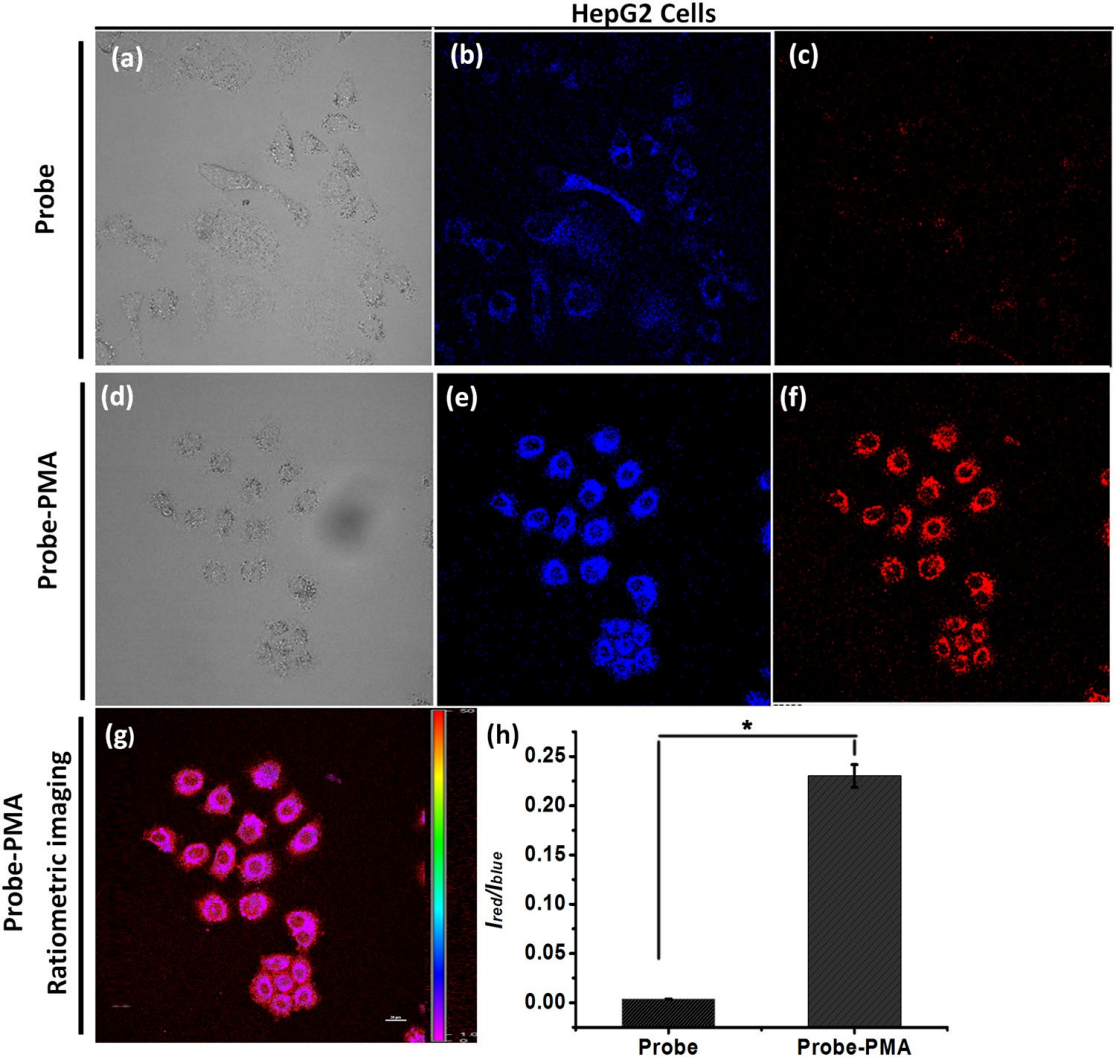
(**a**) Bright-field image of cells untreated with PMA, (**b**) Images of cells untreated with PMA in the blue emission channel (λ_ex_ = 405 nm, λ_em_ = 425–475 nm); (**c**) Images of cells untreated with PMA in the red emission channel (λ_ex_ = 405 nm, λ_em_ = 570–620 nm); (**d**) Bright-field images of cells treated with PMA; (**e**) Images of cells treated with PMA in the blue emission channel (λ_ex_ = 405 nm, λ_em_ = 425–475 nm); (**f**) Images of cells treated with PMA in the red emission channel (λ_ex_ = 405 nm, λ_em_ = 570–620 nm); (**g**) Ratiometric images of cells untreated with PMA. (**h**) The ratio of emission intensities profiles in HepG2 cells untreated and treated with PMA. Scale bar = 20 μm. Statistical analyses were performed with a Student’s *t*-test (*n* = 4). *P < 0.001 and the error bars represent standard deviation (±S.D.).

It has been known for many years that H_2_O_2_ plays a major role in the progression of human illnesses, perhaps most importantly, cancer^34^. To demonstrate the probe **TPE-TLE** ratiometric imaging exogenous H_2_O_2_, the living cancer cells HepG2 and HepG2 treated with PMA were prepared. Imaging results indicated that cells untreated with PMA gave a strong blue fluorescence signal and a weak red signal in blue and red channels, respectively (Fig. 8a–c). However, images of cells treated with PMA results demonstrated that cells displayed a strong blue emission (Fig. 8e) and a strong red emission (Fig. 8f). Similarly with RAW 264.7 macrophages, ratiometric fluorescence imaging picture (Fig. 8g) and fluorescence emission profiles (Fig. 8h) were obtained. Thus, the above results establish that **TPE-TLE** is cell-membrane permeable ratiometric probe with AIE property, and suitable for the ratiometric imaging enogenous H_2_O_2_ in the living HepG2 cells by an aggregation emission method.

Compared to RAW 264.7 macrophages, both channels exhibited stronger fluorescence in living HepG2 cells than RAW 264.7 macrophages in the absence and presence of PMA. Moreover, the **TPE-TLE** probe emitted stronger red fluorescence in HepG2 cells treated with PMA than RAW 264.7 macrophages. Furthermore, the **TPE-TLE** exhibited higher ratio of emission intensities in HepG2 cells treated with PMA than RAW 264.7 macrophages. The above results demonstrated that cancer cells should contain more H_2_O_2_ than macrophages.

To the best of our knowledge, no reports to date have been published on the construction of ratiometric H_2_O_2_ fluorescent probes with AIE property for ratiometric fluorescent imaging endogenously H_2_O_2_ in RAW 264.7 macrophage and HepG2 cells.

## Conclusions

In summary, we introduced a thiazole group and a borate moiety on the TPE-core to afford a novel ratiometric H_2_O_2_ probe **TPE-TLE** with AIE property for the first time. For sensing mechanism of **TPE-TLE**, in the presence of H_2_O_2_, the oxidation reaction of H_2_O_2_ to the borate moiety will provide TPE-TLE-O, which will decrease aggregation fluorescence signal of **TPE-TLE** to induce aggregation fluorescence signal of TPE-TLE-O. Furthermore, the **TPE-TLE** exhibits excellent properties including the well-resolved emission peaks, high sensitivity, high selectivity, low cytotoxicity, and good cell-membrane permeability. The above mechanism and attributes enable us to demonstrate, for the first time, the ratiometric imaging of endogenously produced H_2_O_2_ in macrophages and cancer cells. Moreover, by comparing to fluorescence and ratio of emission intensities of both cells, it is firstly found that cancer cells should contain much more endogenous H_2_O_2_ than macrophages. We expect that **TPE-TLE** will be useful fluorescent platform for the development of a variety of ratiometric fluorescent probes with AIE property, and further achieve unique biological applications.

## Methods

### General procedure for the spectral measurement

The stock solution of the probe **TPE-TLE** was prepared at 1 mM in DMSO. The PBS (pH = 7.4) solutions with 50% DMSO was prepared. The solutions of various testing species were prepared from NaCl, KCl, CaCl_2_, glutathione (GSH), H_2_O_2_, homocysteine (Hcy), cysteine (Cys), NaNO_2_, NaOH, t-butylhydroperoxide, NaClO, in the twice-distilled water. The test solution of the probe **TPE-TLE** (5 μM) were 50% PBS buffer solution (containing 50% DMF) and 90% PBS buffer solution (containing 10% DMF). The resulting solution was shaken before measuring the spectra. For titration and selectivity experiments of probe, both experiments were carried out using excitation wavelength of 405 nm, and the excitation and emission slit widths of the spectral measurement were 5 and 5 nm, respectively.

### Quantum yields

The fluorescence quantum yields would be calculated by the following formula (1):

1 $${{\rm{\Phi }}}_{s}={{\rm{\Phi }}}_{r}(\frac{{{\rm{A}}}_{r}}{{{\rm{A}}}_{s}})(\frac{{{n}_{s}}^{2}}{{{n}_{r}}^{2}})\frac{{{\rm{I}}}_{s}}{{{\rm{I}}}_{r}}$$

In this formula, *s* and *r* stand for the sample and the reference, respectively*. Ф* stands for quantum yield; *F* and A stands for the integrated emission intensity and the absorbance, respectively; *n* is refractive index^35^.

### Cytotoxicity assay

Living RAW264.7 cells were cultivated in per well of 96-well plate. After 24 h, the culture medium was changed using new culture medium containing different concentration (0 μM, 1 μM, 5 μM, 10 μM, 20 μM) **TPE-TLE**. After 8 h, the culture medium and the excess probes were removed, and then 10 μL MTT (5 mg/mL in PBS) was added to the above medium. Subsequently, the culture medium was removed, and 100 μL DMSO was added to the 96-well plate in order to dissolve the formazan crystal product. The plate containing Living RAW264.7 cells was shaken for 10 min, and then the plate containing RAW264.7 cells was measured at 490 nm using the microplate reader. OD_490_ sample denotes the RAW264.7 cells incubated with the probe **TPE-TLE** at different incubation times, OD_490_ control denotes the RAW264.7 cells without the probe **TPE-TLE**, OD_490_ blank denotes the wells containing only the culture medium. The cell viability would be calculated by the following formula: Cells viability (%) = (OD_490 sample_ − OD_490 blank_)/(OD_490 control_ − OD_490 blank_) × 100%^36, 37^.

### Cells culture

Living RAW264.7 macrophages were prepared in H-DMEM (Dulbecco’s Modified Eagle’s Medium, High Glucose) supplemented with 10% fetal bovine serum in a 5% CO_2_ incubator at 37 °C. Before the living cells imaging experiments, living RAW264.7 cells were seeded confocal dish (density of cells was 1 × 10^5^/mL). The cells were placed on glass coverslips and allowed to adhere for 48 h. Cells imaging experiments could be carried out as soon as the cells reached about 70% confluence.

Firstly, control experiment was further carried out. The culture medium of the cells was added to a fresh medium containing 50.0 μg/mL 12-myristate-13-acetate (PMA) and incubated for 3 h. Secondly, the medium was removed and washed three times with PBS in order to remove the excess PMA. After that, 1 mL of the medium containing 8 μM **TPE-TLE** was added and further incubated at 37 °C for 30 min. Finally, the confocal imaging was carried out in the blue (λ_ex_ = 405 nm, λ_em_ = 425–475 nm) and green channels (λ_ex_ = 405 nm, λ_em_ = 570–620 nm) using Nikon AMP1 confocal microcopy.

### Synthesis of 1

The compound was synthesized by refer literature^38^.

### Synthesis of TPE-TLE-O

In a round-bottomed flask (25 mL) equipped with a magnetic stirrer, a solution of the compound 1 (0.37, 1.0 mmol) and 2-aminothiophenol (0.15 g, 1.2 mmol) in methanol (10 mL) was prepared. Then, 30% H_2_O_2_ (6.0 mmol) and 37% HCl (3.0 mmol) were added and the mixture was stirred at room temperature for 2 h. The mixture was quenched by adding H_2_O (10 mL), extracted with EtOAc (3 5 mL), and the combined extracts were dried (Na_2_SO_4_). The corresponding benzothiazoles were obtained after removal of solvents and purified by silica gel chromatography (eluent: n-hexane/EtOAc = 4:1). The yield of **TPE-TLE-O** was 65%. ^1^H NMR (400 MHz, CDCl_3_) *δ* 12.47 (s, 1 H), 7.99–7.94 (m, 1 H), 7.85 (dd, *J* = 8.0, 1.1 Hz, 1 H), 7.50 (m, 1 H), 7.42–7.35 (m, 2 H), 7.20–7.02 (m, 16 H), 6.88 (d, *J* = 8.6 Hz, 1 H). ^13^C NMR (101 MHz, DMSO-*d6*) *δ* 155.39, 151.77, 143.78, 143.45, 140.09, 135.41, 135.07, 131.26, 131.12, 128.44, 128.38, 128.29, 127.16, 127.03, 126.94, 125.52, 122.54, 122.41, 116.95.

### Synthesis of TPE-TLE

Compound **2** (0.24 g, 0.5 mmol) was added into a flask containing a mixture of 4-bromomethylphenyl boronic acid (0.15 g, 0.5 mmol), K_2_CO_3_ (0.07 g, 0.5 mmol), and 10 mL of DMF with nitrogen at room temperature for 6 h, then poured into H_2_O (500 mL) and extracted with EtOAc The organic phase was separated, dried with MgSO_4_, and removed by vacuum distillation. The product was obtained as a yellow solid with a yield of 60% after purified by column chromatography with ethyl acetate/petroleum ether (2:1, v/v) as eluent. Melting point: 142–146 °C. ^1^H NMR (400 MHz, DMSO-*d6*) *δ* 8.10 (s, 1 H), 8.05–8.07 (d, *J* = 8.0 Hz, 1 H), 7.95–7.97 (d, *J* = 8.4 Hz, 1 H), 7.71–7.73 (d, *J* = 7.6 Hz, 2 H),7.56–7.58(d, *J* = 8.0 Hz, 2 H), 7.48–7.57 (t, *J* = 7.8 Hz, 1 H), 7.38–7.46 (t, *J* = 7.4 Hz, 1 H), 6.99–7.16 (m, 17 H), 5.40 (s, 2 H), 1.30 (s, 12 H). ^13^C NMR (101 MHz, DMSO) δ 162.21, 154.88, 151.80, 143.63, 143.55, 143.37, 141.36, 139.89, 139.80, 136.73, 135.77, 135.15, 135.08, 131.30, 131.21, 131.11, 128.42, 128.30, 127.96, 127.19, 127.03, 126.71, 125.38, 122.86, 122.25, 121.51, 113.71, 84.19, 70.82, 40.65, 40.44, 40.24, 40.03, 39.82, 39.61, 39.40, 25.16. HRMS (ESI) (m/z): [M + H]^+^ calcd for C_46_H_40_BNO_3_S: 698.3000, found, 698.3001.

## Electronic supplementary material

Supporting information
