# Supplementary material for: Ratiometric fluorescent probe with AIE property for monitoring endogenous hydrogen peroxide in macrophages and cancer cells
Source: Sci Rep. 2017 Aug 4;7:7293. doi: 10.1038/s41598-017-07465-5 (PMC5544719; doi:10.1038/s41598-017-07465-5)
Supplement: Supplementary file 1 — Supporting information [file 41598_2017_7465_MOESM1_ESM.doc]

*Supporting Information*

**Ratiometric fluorescent probe with AIE property for monitoring endogenous hydrogen peroxide in macrophages and cancer cells**

Yong Liu‡^a^, Jing Nie‡^b^, Jie Niu^a^, Fangfang Meng^a^, Weiying Lin ^a^,*

*^a^ Institute of Fluorescent Probes for Biological Imaging, School of Chemistry and Chemical Engineering, School of Materials Science and Engineering, University of Jinan, Shandong 250022, P. R. China.*

*E-mail:* [*weiyinglin2013@163.com*](mailto:weiyinglin2013@163.com)

*^b^ School of Chemical Engineering & Technology, China University of Mining and Technology, Xuzhou, Jiangsu, 221116, P.R. China.*

*‡ Liu and Nie made equal contributions to this work.*

Table of contents

Page

Table S1.………………………………………………………..............................S4

Figs S1-S2, Table S2………………………………………………………….…...S5

Figs S3-S4…………………………………………….……………………….......S6

Figs S5-S6………………………………………………………………………....S7

Figs S7-S8…………………………………………………………………………S8

Figs. S9-S10…………………………………………….………………………....S9

References…………………………………………….……………………………S10

**Materials**

2-aminothiophenol and 4-bromomethylphenyl boronic acid were purchased from J&K Chemical (Beijing, China). Phosphate Buffered Saline (PBS), Dulbecco’s Modified Eagle’s Medium, High Glucose and 3-(4,5-dimethylthiazol-2-yl)-2,5-diphenyltetrazolium bromide (MTT) were obtained from Seikagaku Corporation (Japan). RAW 264.7 cells, HepG2 cells and calf serum were obtained from the College of life science, Nankai University (Tianjin, China).The solvents used in the spectral measurement are of chromatographic grade. All reagents were purchased from commercial suppliers and used without further purification. Solvents were purified by standard methods prior to use. Twice-distilled water was used throughout all experiments. TLC analysis was performed on silica gel plates and column chromatography was conducted over silica gel (mesh 200–300), both of which were purchased from the Qingdao Ocean Chemicals.

**Measurements**

Mass spectra were performed using an LCQ Advantage ion trap mass spectrometer from Thermo Finnigan or Agilent 1100 HPLC/MSD spectrometer. NMR spectra were recorded on an AVANCE III 400 MHz Digital NMR spectrometer. Electronic absorption spectra were obtained on a Labtech UV Power PC spectrometer. Photoluminescent spectra were recorded at room temperature with a HITACHI F4600 fluorescence spectrophotometer. TLC analysis was performed on silica gel plates and column chromatography was conducted over silica gel (mesh 200–300), both of which were obtained from the Qingdao Ocean Chemicals. Fluorescence imaging of the cells was obtained using Nikon A1MP confocal microscopy.

**Table S1.** Properties of the representative hydrogen peroxide probes with AIE property developed and the **TPE-TLE** reported in this work.

| Ref. | Core structure | Stokes shift *in aqueous solution* | Ratiometric imaging | Maximum absorption wavelength and emission wavelength *in aqueous solution* | Application |
| --- | --- | --- | --- | --- | --- |
| **This work** | **TPE-TLE** | 163 nm | Yes | 307/470nm | Ratiometric imaging hydrogen peroxide |
| [^1^](#_ENREF_1) | TPE-BO | 100 nm | No | 400/500nm | Rapid-Response detecting hydrogen peroxide in living  Cells |
| [^2^](#_ENREF_2) | TPE-DABA  TPE-DABF | _ | No | -/576nm | Highly sensitive detection of hydrogen peroxide |
| [^3^](#_ENREF_3) | Compound 1 | _ | No | -/510nm | Highly sensitive detection of hydrogen peroxide  and glucose |
| [^4^](#_ENREF_4) | TPE-HPro | _ | No | _ | Highly sensitive detection of hydrogen peroxide  and glucose |
| [^5^](#_ENREF_5) | TPA-PR-BOR | - | No | -/500nm | Highly sensitive detection of hydrogen peroxide |

**Table S2.** The photophysical properties of **TPE-PLE**

| Slovents |   ^a^λ/nm |   ^b^λ/nm | Stokes shifts |  ^c^ϕ/% |
| --- | --- | --- | --- | --- |
| DMF | 306 | 420 | 114 | 11.70 |
| DMSO | 307 | 430 | 123 | 5.14 |
| EtOH | 308 | 430 | 122 | 1.76 |
| CHCl_3_ | 306 | 425 | 119 | 3.60 |
| H_2_O | 307 | 470 | 163 | 60.68 |
| PBS | 324 | 470 | 146 | 62.26 |

^a^Maximum absorption wavelength (nm). ^b^Maximum emission wavelength (nm). c is fluorescence quantum yield (error limit: 8%) determined by using fluorescein (ϕ= 0.95) in aqueous NaOH (pH = 13) as the standard.


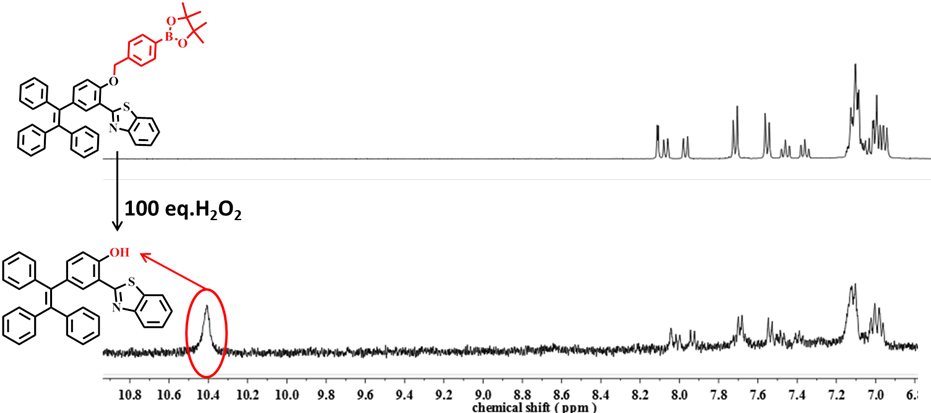


**Fig S1**. The ^1^H NMR spectrum of the hydroxy peak of **TPE-TLE** in the absence and presence of H_2_O_2_ (100 equiv) in *d*_6_ - DMSO/D_2_O (4:1).


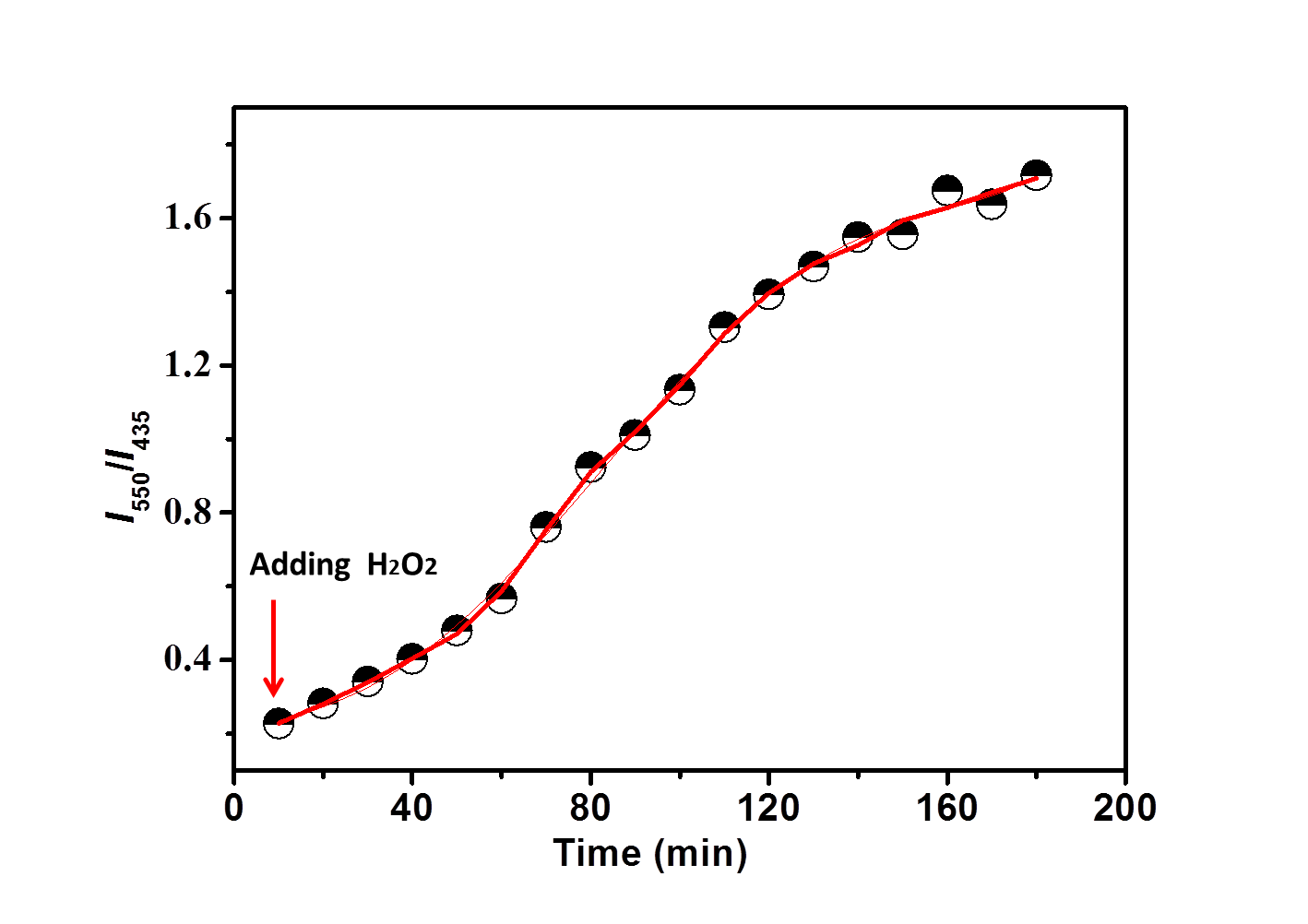


**Fig S2*.*** Time course of the fluorescence intensity of **TPE-TLE** (5 μM) at *I*_550_/*I*_435_ after adding H_2_O_2_. λ_ex_ = 380 nm.

**
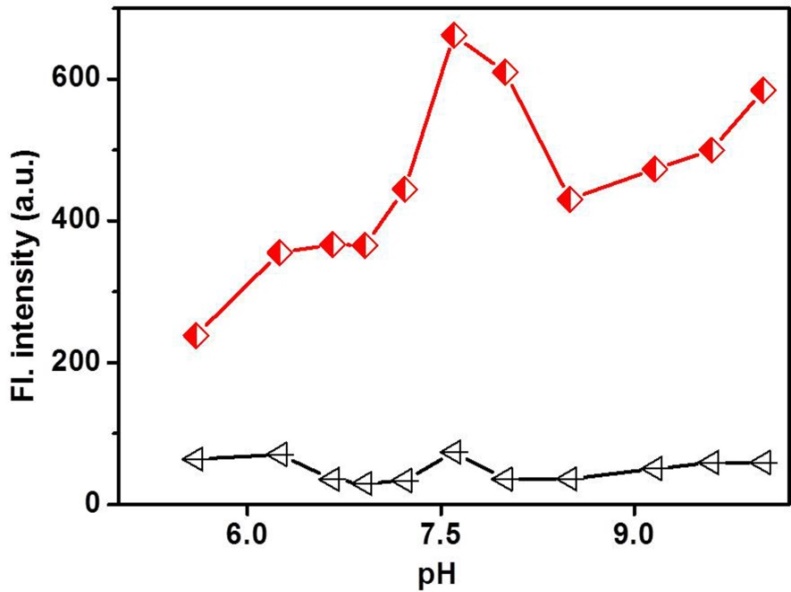
**

**Fig S3.** The fluorescence emission intensity changes (at 575 nm) of **TPE-TLE** before and after upon addition of H_2_O_2_ at different pH PBS buffer solution. λ_ex_ = 380 nm.


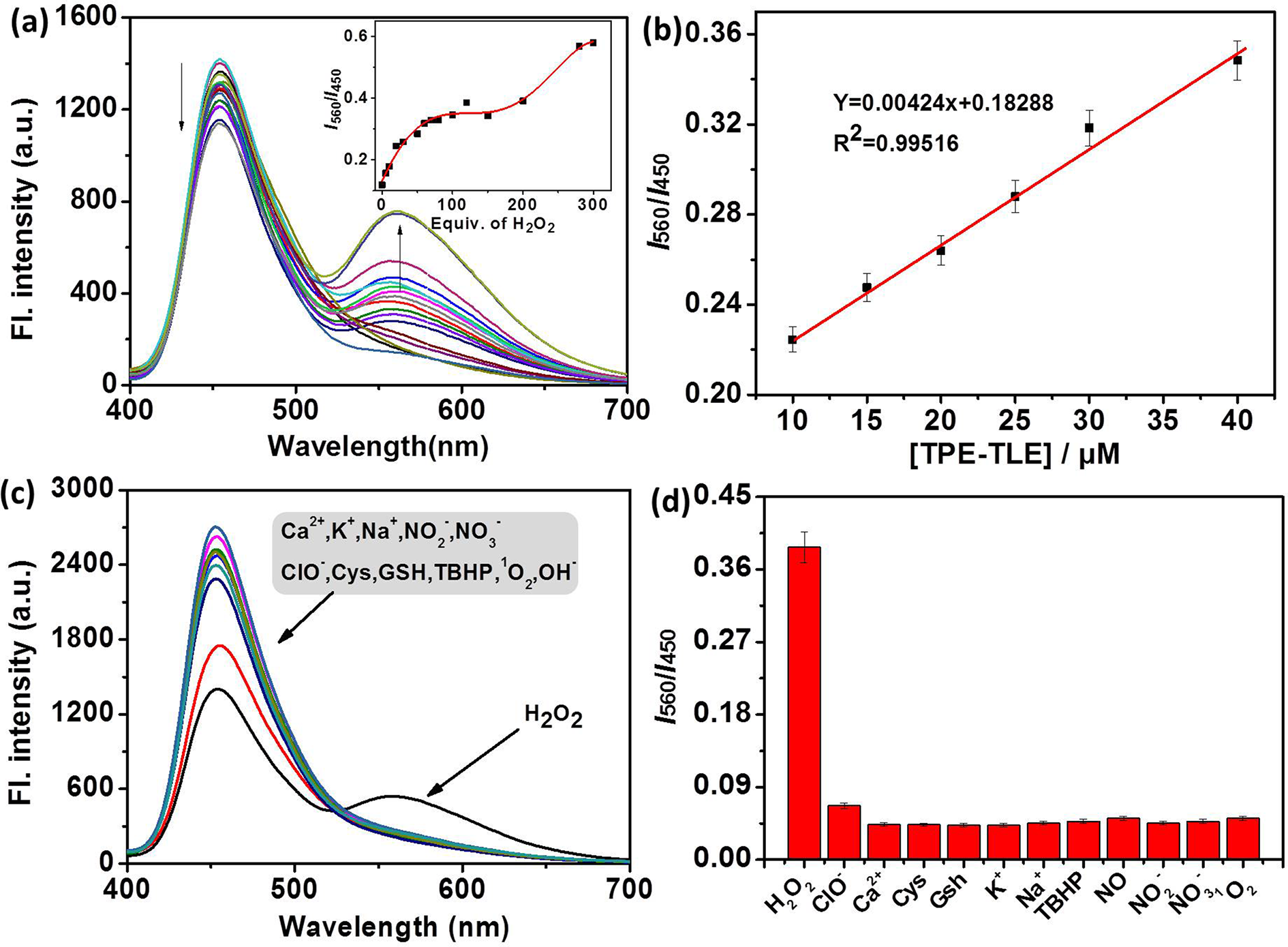


Fig S4. (a) Fluorescence spectra of TPE-TLE (5.0 μM) in pH 7.4 PBS buffer solution (containing 10% DMF) with the addition of H_2_O_2_. Inset: the fluorescence intensity changes at *I*_560_/*I*_450_ of TPE-TLE with the amount of H_2_O_2_. (b) Normalized response of the fluorescence signal by changing the concentration of H_2_O_2_. λ_ex_ = 380 nm. (c) Fluorescence spectra of TPE-TLE (5 μM) in the presence of various relevant analytes. (d) Fluorescence responses of TPE-TLE (5 μM) at 560 nm in the presence of various relevant analytes. The concentrations of the representative analytes are: amino acids, 1 mM; GSH, 2 mM; cations and anions, 3 mM; reactive oxygen and nitrogen species, 0.2 mM.


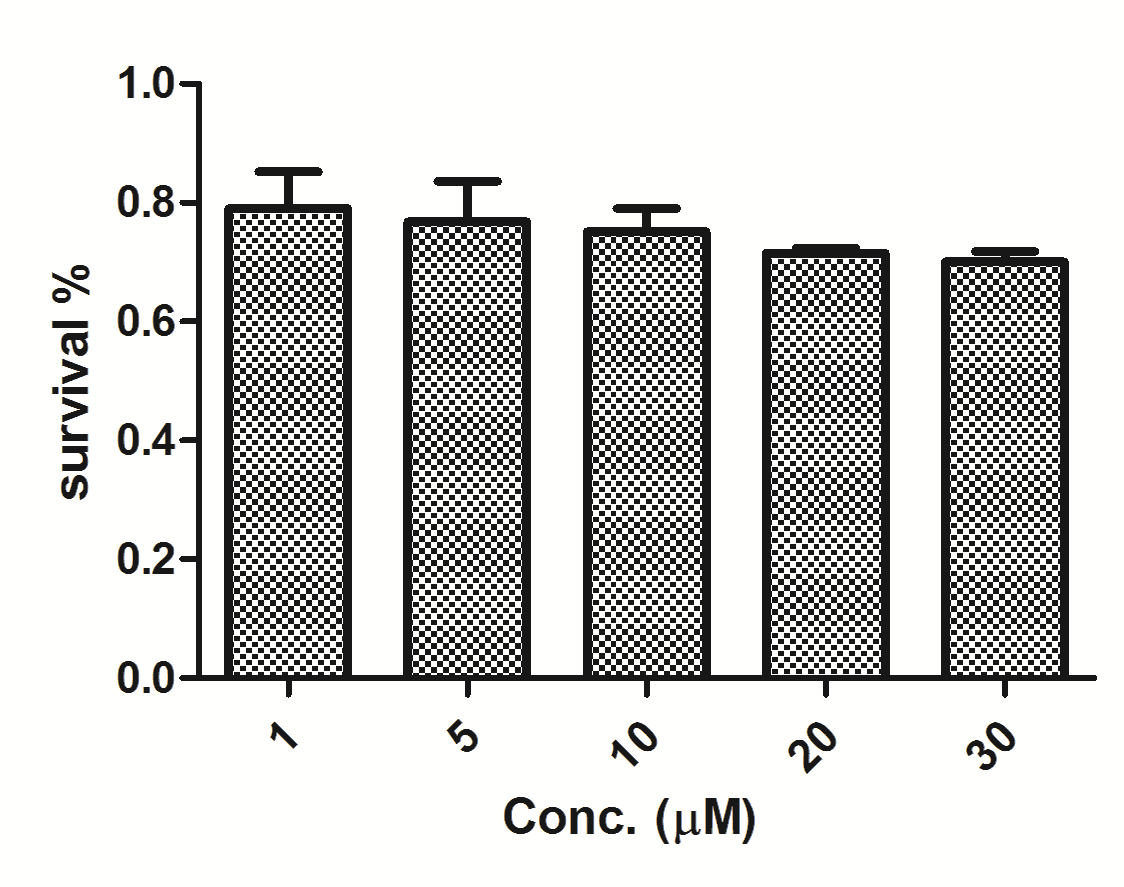


**Fig S5.** Cytotoxicity data of **TPE-TLE**


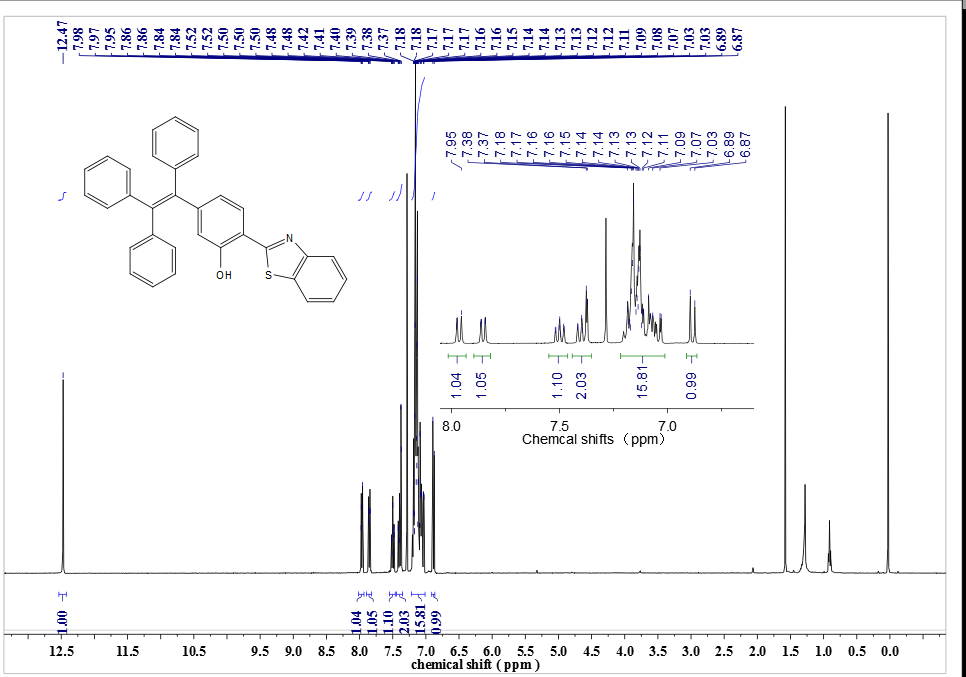


**Fig S6.** ^1^H NMR spectrum of the compound **2**


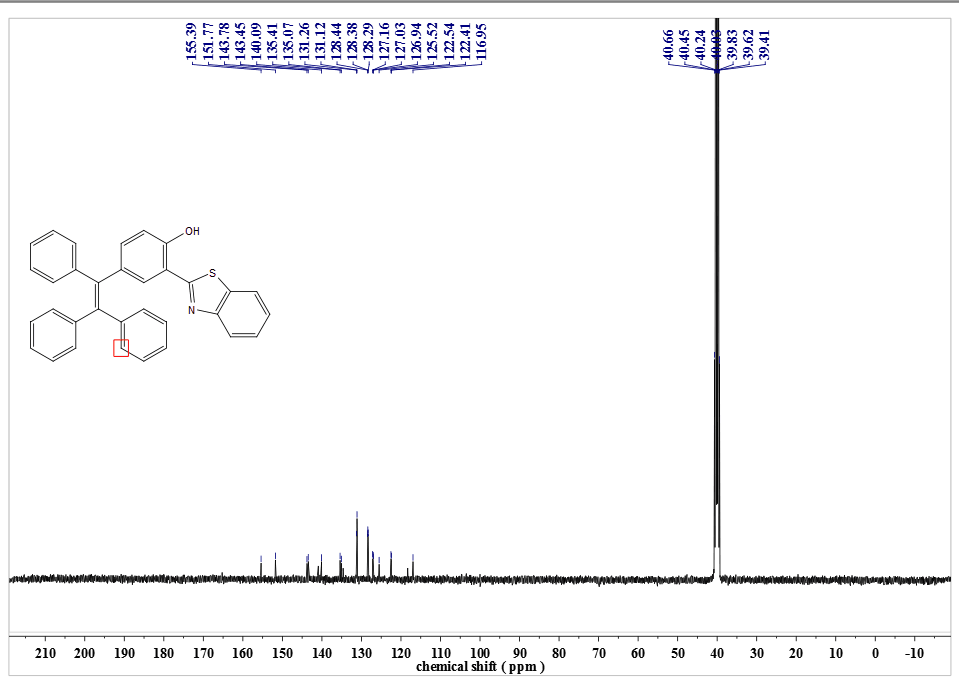


**Fig S7.** ^13^C NMR spectrum of the compound **2**


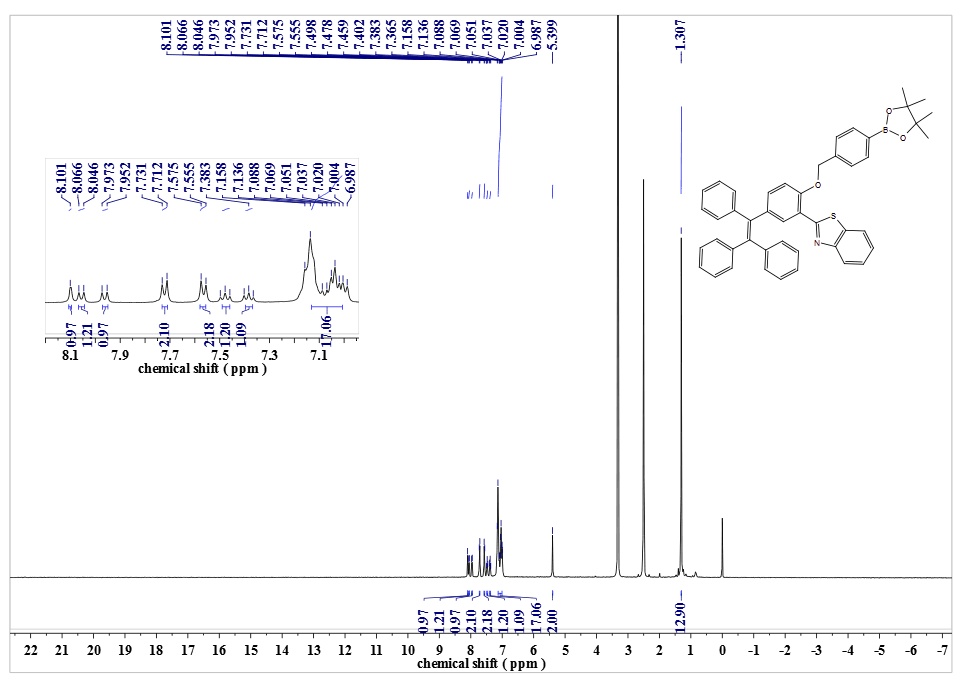


**Fig S8.** ^1^H NMR spectrum of **TPE-TLE**


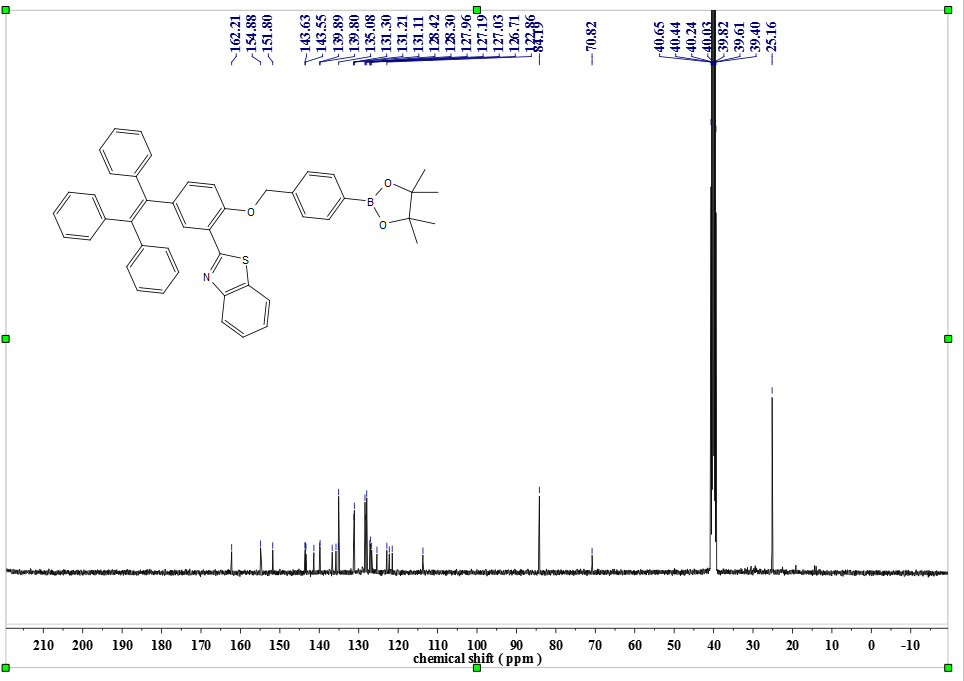


**Fig S9.** ^13^C NMR spectrum of **TPE-TLE**


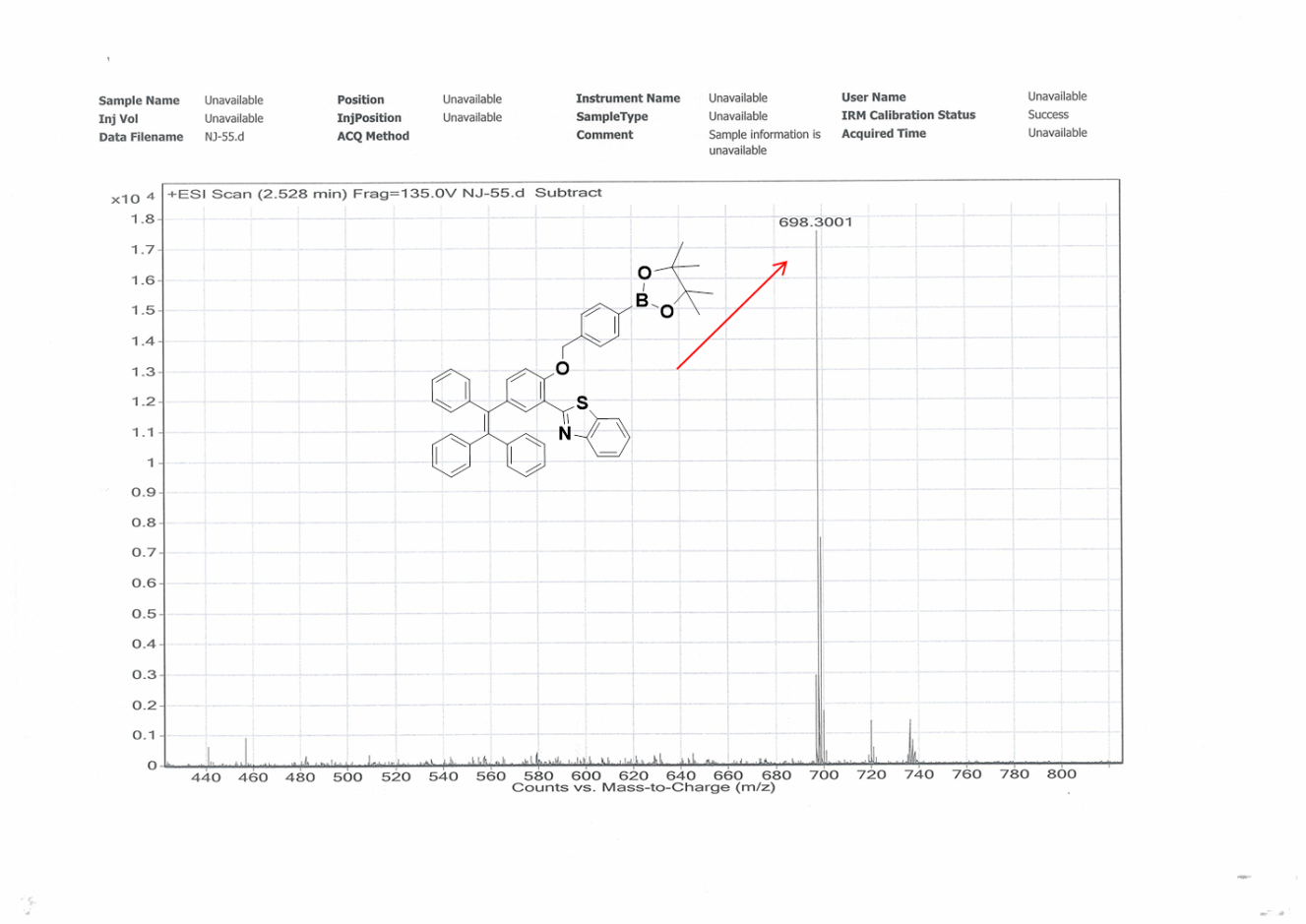


**Fig S10.** H RMS spectrum of **TPE-TLE**

**References**

1. Zhang, W. *et al*. Rapid-response fluorescent probe for hydrogen peroxide in living cells based on increased polarity of C–B bonds. *Anal. Chem*. **87**, 9825-9828 (2015).

2. Liu, G. J. *et al*. A dialdehyde–diboronate-functionalized AIE luminogen: design, synthesis and application in the detection of hydrogen peroxide. *Chem. Commun*. **52**, 10233-10236 (2016).

3. Hu, F., Huang, Y., Zhang, G., Zhao, R. & Zhang, D. A highly selective fluorescence turn-on detection of hydrogen peroxide and D-glucose based on the aggregation/deaggregation of a modified tetraphenylethylene. *Tetrahedron Lett*. **55**, 1471-1474 (2014).

4. Song, Z. *et al*. An AIE-active fluorescence turn-on bioprobe mediated by hydrogen-bonding interaction for highly sensitive detection of hydrogen peroxide and glucose. *Chem. Commun*. **52**, 10076-10079 (2016).

5. He, C. *et al*. A highly efficient fluorescent sensor of explosive peroxide vapor via ZnO nanorod array catalyzed deboronation of pyrenyl borate. *Chem. Commun.* **48**, 5739-5741 (2012).
